# Efficacy and safety of dapsone in adult immune thrombocytopenia: a systematic review and meta-analysis

**DOI:** 10.1186/s40001-025-03427-0

**Published:** 2025-12-11

**Authors:** Iftikhar Khan, Soban Ali Qasim, Shree Rath, Haris Mumtaz Malik, Sozen Rehman, Sara Aleem, Hammad Javaid, Ahmad Omar Saleh, Syed Ahtisham Halim, Kamil Ahmad Kamil

**Affiliations:** 1 FMH College of Medicine and Dentistry, Lahore, Pakistan; 2 Multan Medical and Dental College, Multan, Pakistan; 3 All India Institute of Medical Sciences, Bhubaneswar, India; 4 Rawalpindi Medical University, Rawalpindi, Pakistan; 5 Khyber Girls Medical College, Peshawar, Pakistan; 6 Sialkot Medical College, Sialkot, Sialkot, Pakistan; 7 King Edward Medical University, Lahore, Pakistan; 8 The University of Jordan, Amman, Jordan; 9 Jinnah Sindh Medical University, Karachi, Pakistan; 10 Internal Medicine Department, Mirwais Regional Hospital, Kandahar, Afghanistan

**Keywords:** Immune Thrombocytopenia (ITP), Dapsone, Platelet response, Hemolytic anemia, Thrombopoietin receptor agonists

## Abstract

**Background:**

Immune thrombocytopenia (ITP) is an autoimmune condition often managed with corticosteroids, intravenous immunoglobulins, or thrombopoietin receptor agonists. In settings where these therapies are inaccessible or contraindicated, dapsone an older antimicrobial agent with immunomodulatory effects has been proposed as an alternative, though its comparative effectiveness and safety profile remain inadequately defined.

**Objectives:**

This study systematically reviews and synthesizes existing data to assess the efficacy and tolerability of dapsone in adult patients with ITP.

**Methods:**

Following PRISMA guidelines, a literature search across PubMed, Embase, and Cochrane databases identified observational studies and interventional trials evaluating dapsone in ITP, including comparisons with standard treatments. Primary outcomes included complete and overall response rates, hemoglobin variation, relapse frequency, and adverse effects. Risk of bias was evaluated using the Newcastle–Ottawa Scale and Cochrane tools. Meta-analysis was conducted using RevMan and R (meta/metafor).

**Results:**

Four eligible studies encompassing over 270 patients were analyzed. The pooled complete response rate was 28%, while the overall response rate reached 47%. Dapsone use was associated with a modest decline in hemoglobin levels with a mean decrease of 1.74 g/dL, and relapse occurred in approximately 21% of patients. Adverse effects most commonly hemolytic anemia and methemoglobinemia were reported in 18% of patients, though generally not severe. Heterogeneity was explored through leave-one-out sensitivity analyses.

**Conclusions:**

Dapsone demonstrates moderate efficacy and an acceptable safety profile in adult ITP patients, especially in resource-limited contexts. While it may not match thrombopoietin agonists in preventing relapse, its low cost and oral administration offer practical advantages. Further large-scale randomized trials are warranted to clarify its role in ITP management.

**Supplementary Information:**

The online version contains supplementary material available at 10.1186/s40001-025-03427-0.

## Introduction

Immune thrombocytopenia (ITP) is an autoimmune condition in which antibodies bind to platelets and promote their destruction, while T cells directly damage megakaryocytes. This leads to reduced platelet counts and an increased risk of bleeding [1]. Although anyone can be affected, ITP is more common in older adults [1]. In the absence of other causes of thrombocytopenia, a platelet count below 100 × 10^9^/L is required for diagnosis [2].

First-line therapies include corticosteroids, intravenous immunoglobulin (IVIg), and anti-D. These achieve initial responses in 70–80% of patients within a few days; however, relapse is common once treatment is tapered [3]. Thrombopoietin receptor agonists (TPO-RAs) are now widely recommended as second-line therapy for corticosteroid-dependent or persistent ITP [4]. International guidelines, including those of the American Society of Hematology, recommend TPO-RAs as the preferred second-line option, with rituximab and splenectomy as alternatives [5]. Fostamatinib has also been included in more recent guideline updates as an additional option [6].

In regions where these agents are unavailable or unaffordable, such as parts of Europe, dapsone continues to be used off-label and is mentioned in some treatment guidelines [7]. Although its role is less prominent than that of TPO-RAs or rituximab, dapsone remains an alternative option in certain cases or resource-limited settings [4]. Moreover, some studies have reported that dapsone has been used in patient’s refractory to TPO-RAs, rituximab, or even after splenectomy, suggesting a potential role beyond strictly resource-restricted areas [8].

Dapsone is a low-cost drug with antimicrobial, anti-inflammatory, and immunomodulatory properties [9]. Several studies have evaluated its efficacy in raising platelet counts and reducing bleeding in patients with ITP. In the largest study to date, approximately 50% of patients responded to a daily dose of 75–100 mg, with a median time to response of 21 days [10]. However, compared with TPO-RAs and rituximab—which have demonstrated durable responses and favorable safety profiles in randomized trials—the evidence supporting dapsone is limited to small, mostly observational studies [5, 6].

Although these findings suggest that dapsone may represent a safe and affordable treatment for chronic ITP, its relative effectiveness compared with standard therapies such as corticosteroids, IVIg, and TPO-RAs remains uncertain. In addition, multiple cost-effectiveness analyses have shown that while TPO-RAs are clinically effective, they impose a substantial economic burden, limiting their widespread use in many healthcare systems [11]. Given the economic challenges associated with ITP treatment and restricted access to novel agents in several regions, a systematic review comparing dapsone with standard second-line therapies is both clinically relevant and timely.

## Methodology

Our meta-analysis was conducted according to the guidelines provided in the Cochrane Handbook for Systemic Reviews of interventions [12] and reported according to the Preferred Reporting items for Systemic Reviews and Meta-Analysis (PRISMA) statement [1].

### Data sources and searches

A comprehensive literature search was performed using multiple electronic databases, including Medline and Embase *Cochrane Library, and ClinicalTrials.gov*. The following terms (“Immune thrombocytopenia OR ITP” AND “Dapsone” AND “Corticosteroids OR IVIG”) were used as either Medical Subject Heading (MeSH) terms or keywords. The search was limited to English-language publications from their inception to March 2025. In addition, reference lists of included studies and similar systemic reviews were manually screened to identify any pertinent studies, a detailed search strategy of the included studies is available in Supplementary Table S1.

### Study selection

Studies were included in the analysis based on predefined eligibility criteria. Specifically, we considered observational studies involving adult patients with immune thrombocytopenia (ITP). The studies had to compare dapsone with the standard treatment of ITP. We excluded studies that were reviews, editorials, case reports, case series, or animal studies. In addition, studies lacking control or comparison groups were not included in the analysis.

### Screening and data screening

We used Rayyan to narrow down and remove duplicates of all the articles yielded by our online search. Two authors independently (A.O.S., and S.A.H.) performed a screening of titles and abstracts to exclude all irrelevant articles. Full-text screening in accordance with our eligibility criteria was then performed on the remaining studies. Any discrepancies over the selection of studies were settled by a third author (I.K.).

### Data extraction

Relevant data were extracted into a pre-piloted Excel spreadsheet which included author name, publication year, study design, sample size, age, gender, BMI, ITP duration, baseline mean platelet count and baseline hemoglobin level. The primary outcomes were complete response, overall response rate, partial response, change in hemoglobin, relapse rate, and incidence of adverse events. In our analysis, “response” was defined according to the criteria used in each individual study. In most studies, overall response was defined as achieving a platelet count > 30 × 10^9^/L (and at least doubling of the baseline count) without bleeding, while complete response was typically defined as a platelet count > 100 × 10^9^/L.

### Risk-of-bias assessment

The Newcastle Ottawa Scale(NOS) was used to assess the quality of the propensity matched observational studies based on three aspects, the selection of study groups, the comparability of these groups and the establishment of either the exposure or outcome of interest. Two investigators assessed the risk of bias for each of the individual studies as either high, low or some concerns of bias. Any disagreements regarding the risk of bias assessment were settled by a senior investigator.

### Data analysis

All statistical analyses were conducted using Review Manager (RevMan) version 5.4.1). Mantel–Haenszel was used for dichotomous outcomes. Risk ratios (RRs) with 95% confidence intervals (CIs) were calculated using a random effects model to carry out the meta-analysis. Pooled estimates were presented as a forest plot and heterogeneity was rigorously evaluated using the Higgins *I*^2^. Outcomes with high heterogeneity were assessed via Leave-One-Out sensitivity analysis on R-studio. The following packages used “meta” and “metafor”.

## Results

### Search results

**Fig. 1:**
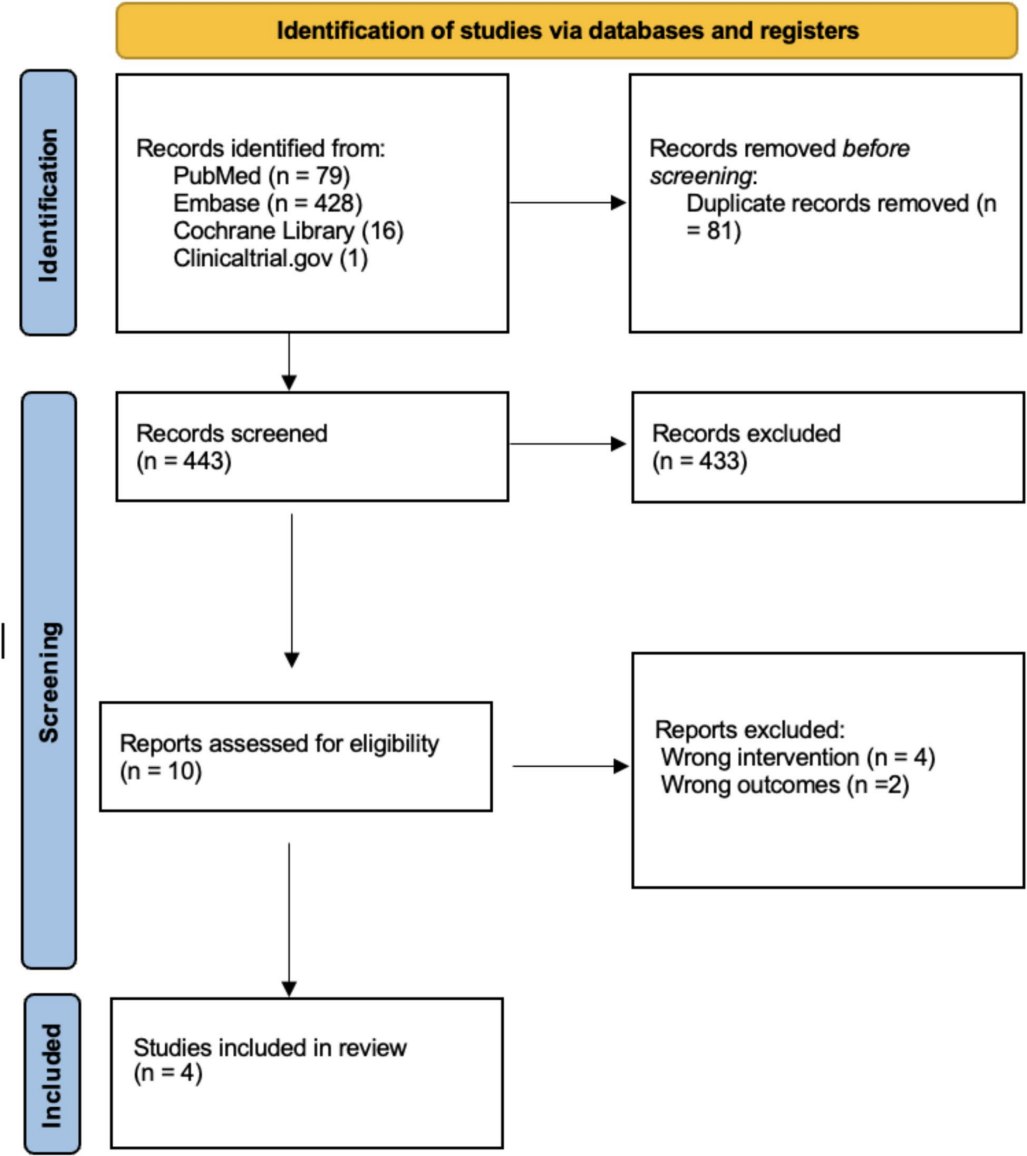
Prisma diagram

The search identified 524 records: 428 from Embase, 79 from PubMed, 16 from Cochrane Library, and 1 from Clinicaltrial.gov. After removing 81 duplicate records, 443 studies remained for the title and abstract screening. Following the screening process, 433 studies were excluded based on title and abstract evaluation due to inappropriate study design, use of interventions other than dapsone, or lack of alignment with our PICO framework. 10 full-text articles were assessed for eligibility and four studies were included in the qualitative and quantitative synthesis [4, 13–15]. The study selection process for inclusion and exclusion was conducted in accordance with PRISMA guidelines and detailed overview of the study selection process is presented in PRISMA flowchart (Fig. 1).

### Baseline characteristics

A total of 270 patients were included across the analyzed studies and the publication years ranged from 2001 to 2024. The detailed baseline characteristics are presented in Table 1.

**Table 1:** Study characteristics

Author, year	Country	Type of study	Age	Sex (Female %)	Sample size	Dosing schedule (median with range)	Sample size	Dosing schedule (median with range)	Sample size
Dutta et al. 2001	India	A prospective (phase1 &2)	28.4 (9.58)	62.5%	8 patients (5F, 3 M)	1 daily oral dose (rechallenge done in some cases)	8 patients (5F, 3 M)	1 daily oral dose (rechallenge done in some cases)	8 patients (5F, 3 M)
Esteve et al. 2017	France	Retrospective cohort	57.1 (34.4–72.2)	64%	42 ITP patients	Not applicable (oral daily dosing)	42 ITP patients	Not applicable (oral daily dosing)	42 ITP patients
Larue et al. 2024	France	Prospective phase III randomized, open-label multicenter trial	48.5 (30.0–64.5) Arm A = 48 [30–65] Arm B = 49 [30–62] Observational study 51 [29–72]”	"Arm A (*n* = 46) = 26 (57%) Arm B (*n* = 47) = 25 (53%) Observational study (*n* = 46) = 24 (52%)”	93 (after exclusions); Observational: 46 (eligible from 127 exposed)	Oral administration daily; not applicable in infusion format	93 (after exclusions); Observational: 46 (eligible from 127 exposed)	Oral administration daily; not applicable in infusion format	93 (after exclusions); Observational: 46 (eligible from 127 exposed)
Colella et al. 2021	Brazil	Retrospective longitudinal cohort	50 (11–84)	73%	122	Not applicable	122	Not applicable	122

### Risk of bias

The multicenter RCT by Larue et al. demonstrated low risk for sequence generation, allocation concealment, detection, attrition, and reporting biases, but had high performance bias due to its open-label design and unclear other bias from early treatment discontinuations (Table S2) [4]. The retrospective case series by Dutta [14] carried high risk across sequence generation, allocation concealment, and performance domains, with unclear detection bias but low attrition and reporting bias (Table S3). The cohort studies (Esteve & Samson; Colella et al.) scored 6–7/9 on the Newcastle–Ottawa Scale, reflecting adequate exposure ascertainment and follow-up but no unexposed comparator and limited confounder control, and demonstrated an overall moderate risk (Tables S2, S3, S4).

### Outcomes

#### Complete response rate

**Fig. 2:**
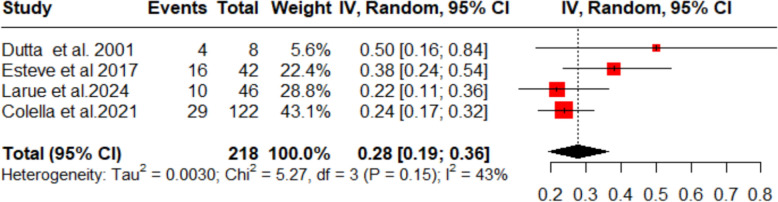
Forest plot (complete response rate)

A pooled complete response rate of 28% (95% CI 19–36%; *I*^2^ = 43%) was observed across four included studies for this dapsone treatment (Fig. 2), meaning around one-third of patients experienced a complete response to the treatment, though moderate heterogeneity was observed sensitivity analysis showed to have no effect on overall heterogeneity value**.**

#### Overall response rate

**Fig. 3:**
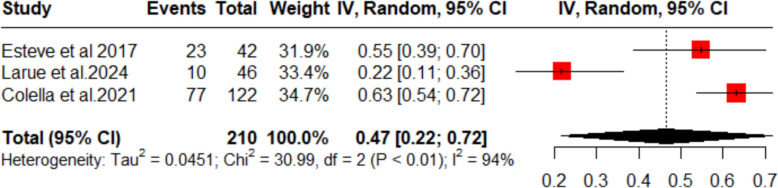
Forest plot (objective response rate)

**Fig. 4:**
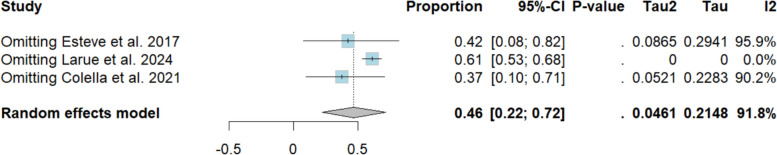
Sensitivity analysis (objective response rate)

The pooled overall response rate (ORR) across three studies was 47% (95% CI 22–72%; Fig. 3). Although there was substantial heterogeneity initially (*I*^2^ = 94%), a sensitivity analysis excluding Larue et al. [4] eliminated all heterogeneity (*I*^2^ = 0%) without changing the overall ORR (Fig. 4).

#### Relapse rate

**Fig. 5:**
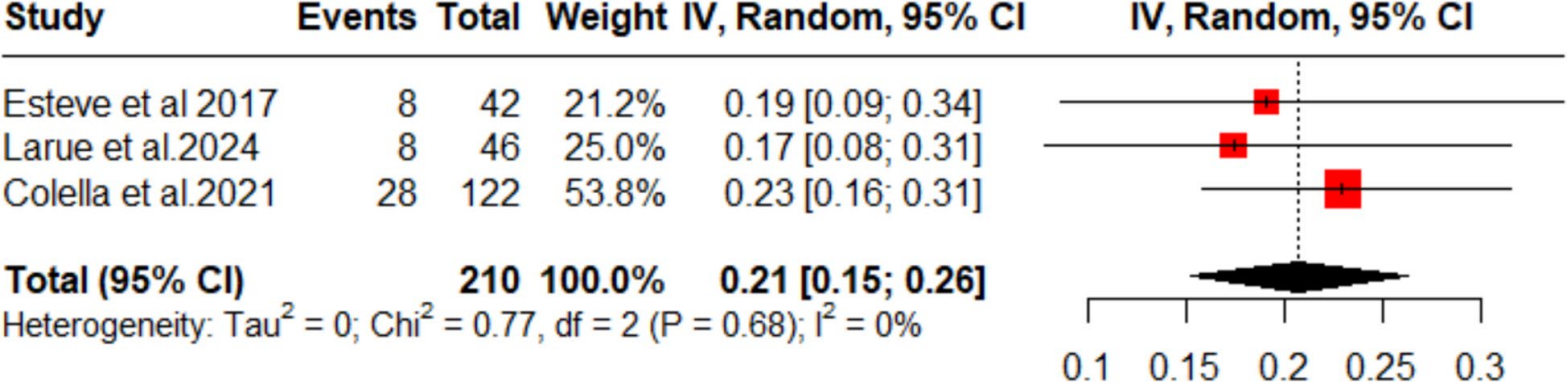
Forest plot (relapse rate)

The pooled analysis of three studies (*n* = 210) treating thrombocytopenic purpura with dapsone demonstrated a relapse rate of 21% (95% CI 15–26%). There was no evidence of between‑study heterogeneity (*I*^2^ = 0%; Fig. 5). These results indicate that about one in five patients may experience disease recurrence following dapsone therapy, underscoring the need for ongoing monitoring after initial remission.

### Mean change in hemoglobin

**Fig. 6:**
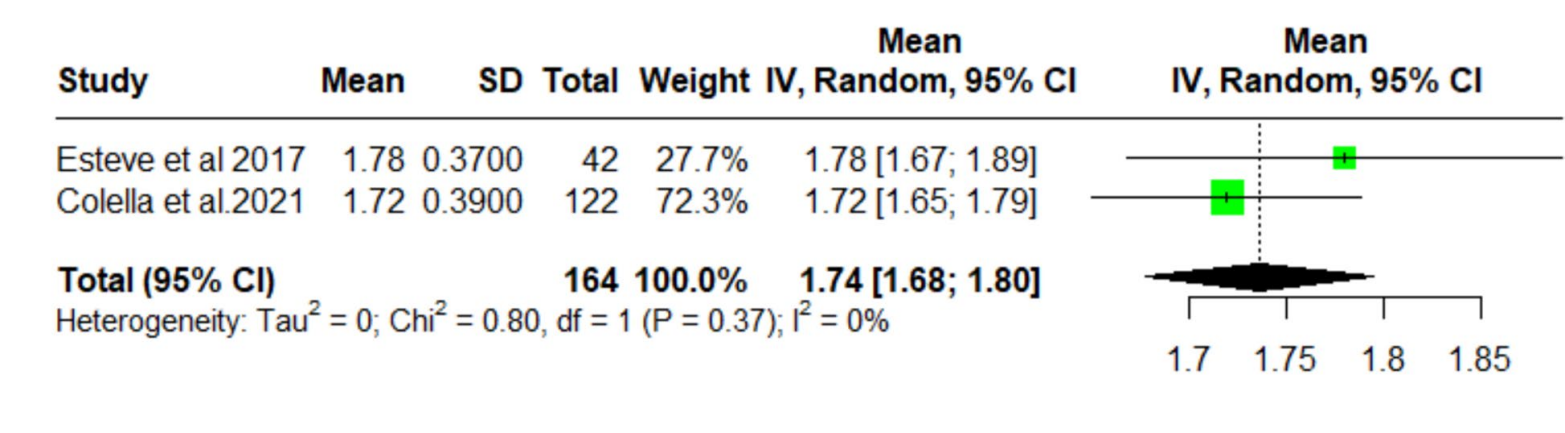
Forest plot (change in hemoglobin)

The two studies (*n* = 164) that measured change in hemoglobin after dapsone treatment demonstrated a pooled decreased mean of 1.74 g/dL (95% CI 1.68–1.80) from baseline, with no detectable heterogeneity between trials (*I*^2^ = 0%; Fig. 6).

### Incidence of adverse events

**Fig. 7:**
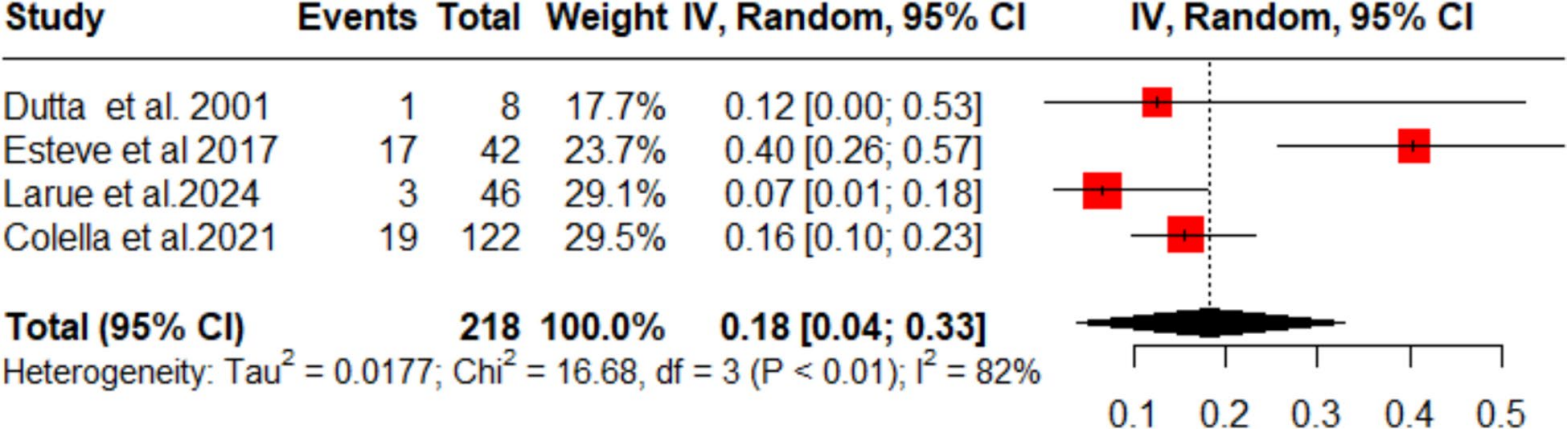
Forest plot (adverse events)

**Fig. 8:**
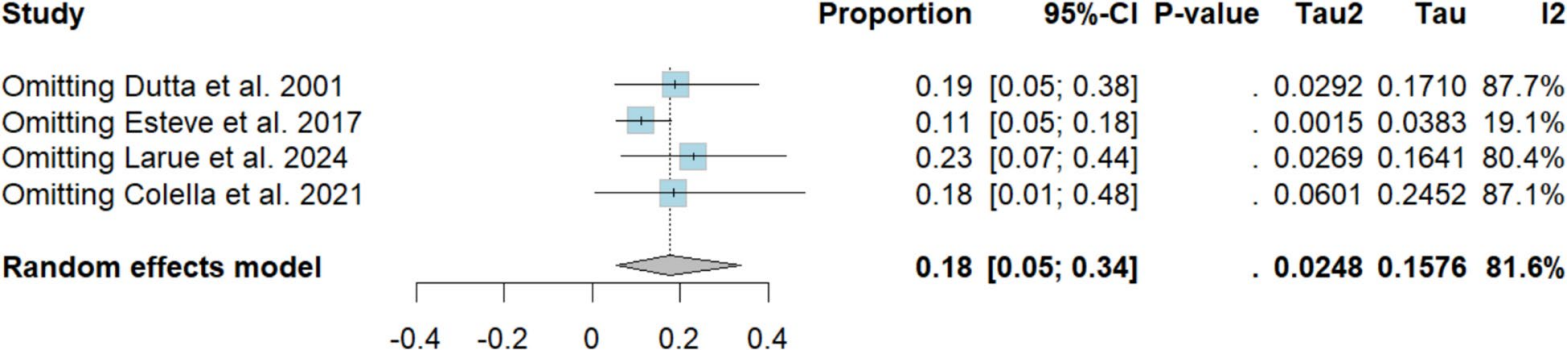
Sensitivity analysis (adverse events)

The pooled incidence of adverse effects among thrombocytopenic purpura patients treated with dapsone was 18% (95% CI 4–33%; *I*^2^ = 82%; Fig. 7). Sensitivity analysis showed that the study by Esteve et al. [15] was responsible for most of the heterogeneity; when this single trial was omitted, the heterogeneity fell to *I*^2^ = 19.1% (Fig. 8). Table of summarized adverse events per study is available in Supplementary Table S5.

## Discussion

This study is a systematic review and a proportionality meta-analysis investigating the efficacy of dapsone in immune thrombocytopenia (ITP). We focused on platelet response, bleeding reduction and overall treatment efficacy. A systematic evaluation of the literature was conducted, allowing us to derive the following important conclusions.

First, this study showed that the proportion of complete response rate (CRR) was 28% in the patients. The overall response rate (ORR) was observed in 47% of the patients. This shows the efficacy of dapsone in ITP patients. This is in line with the report from Bharadwaj et al. [16], which reports an early response in 37.8% of the patients and a partial response in 64.4% of the patients. The complete response on our meta-analysis was reported by Dutta et al. [14] to be 50%; however, the number of participants in this particular study was quite low. On the other hand, the study with the highest weightage in terms of participants was a retrospective analysis by Colella et al. [13] and this study reported it to be 24%. The overall response rate was reported highest by this study, to be 66%. This can be backed by the literature review conducted by Crickx [17], which reports that dapsone can be used in ITP in all conditions except when a rapid response is needed. Therefore, we can conclude that complete response as well as an early response via dapsone are lower as compared to other modalities, such as Thrombopoietin receptor antagonists, but a comparable overall response rate [18]. In addition, this can be compared to overall response of Rituximab [19] which was stated to be 52% but is extremely costly as mentioned in ASH guidelines [20] and Mycophenolate mofetil (MMF) which is stated to be 50–60% [22]. Guidelines also discuss the use of romiplostim and eltrombopag which according to a systematic review and meta-analysis of 2016, showed a response rate of 75% [23]. Dapsone has also been mentioned in the ASH guidelines [21] as a modality and has a slight lower overall response rate as compared to these drugs.

Two of our studies reported a drop in hemoglobin due to dapsone treatment [13, 17]. The total drop in pooled mean hemoglobin was reported to be 1.75 mg/dl (95% confidence interval: 1.68–1.80). This is a notable side effect of dapsone treatment, also reported in patients of kidney transplants by Schaumacher et al. [24]. A study conducted by Naik et al. in India reported that 83.3% of the leprosy patients included in the study reported a fall in hemoglobin from baseline value [25]. This can be explained on the basis that dapsone is known to cause methemoglobinemia. However, this is not reported to be clinically significant [26]. We believe that this study agrees with this, as only a mean drop of 1.7 mg/dl from baseline value can be considered a mild anemia [27]. Anemia is also reported to be a side effect of Rituximab [26], while MMF is reported to cause pure red cell aplasia resulting in severe anemia [28].

All studies reported adverse events with dapsone treatment. A total of 18% patients faced clinically significant adverse events. These include hemolytic anemia, symptomatic methemoglobinemia, gastrointestinal symptoms, skin rash, headache, pruritis and hepatic toxicity [13–15, 29]. Similar adverse effects with the addition of insomnia, blurred vision, paresthesias and neuropathy have been reported by the textbook [28]. We believe that these adverse effects can be attributed to the fact that the mechanism of action of dapsone involve competitive inhibition of dihydropteroate synthase in the folate pathway [28]. As lack of folate results in anemia, competitive inhibition results in hemolysis, resulting in hemolytic anemia [30]. Textbooks have reported similar adverse events for the modalities rituximab and MMF. Romiplostim and eltrombopag have been associated significantly with active bleeding and need for rescue medications compared to placebo [23].

Three of studies reported relapse in ITP after dapsone treatment. This was reported to have occurred in 21% of the pooled population. This relapse is much lower compared to relapses in ITP patients receiving dexamethasone [31]. However, the relapse reported in this study is higher as compared to the relapse of 10% reported by Deng et al. [19], as they used eltrombopag. This shows that dapsone relapse rate is lower than in some more frequently used treatment modalities like dexamethasone, but not as good as thrombopoietin agonists, such as eltrombopag, in terms of ITP relapse.

Treatment cost of 4 week treatment of rituximab is $31,266/- [21]. Similarly, 26 week treatment of eltrombopag and romiplostime were reported to be $66,560 and $91,039, respectively [32]. MMF is also known to be very expensive especially when used on a short term basis, as shown by this cost–utility analysis against azathioprine for proliferative lupus nephritis [33]. Dapsone is much less costly as compared to these modalities and is, therefore, a cost-effective alternative for Immune thrombocytopenia.

There are a few limitations in this study, such as the low number of articles included and the lack of a very large sample size. Therefore, the results of this study cannot be openly generalized and require more original studies and randomized controlled trials for better generalizability of results, Another limitation is that the line of therapy at which dapsone was administered (e.g., first line vs later line) was not consistently reported across studies. However, the major strength of this study was a strict adherence to PRISMA guidelines for meta-analysis. Therefore, we expect future studies to show similar results to our review.

## Conclusion

Our Systematic Review and Proportionality Meta-Analysis has shown that dapsone provides a modest benefit in ITP. While this may be less effective than other therapies in the literature, such as rituximab or MMF, it can serve as a valuable cost-effective alternative in resource-limited settings with a similar adverse effects profile. We believe larger randomized trials are needed, still, to confirm these findings and define its optimal place in therapy.

## Supplementary Information

Supplementary material 1: Table S2: Risk of Bias Assessment. Table S3: Risk of Bias Assessment. Table S4: Risk of Bias Assessment.

## Data Availability

The data used in this article are available upon request from the authors.
