# Supplementary material for: Efficacy and safety of dapsone in adult immune thrombocytopenia: a systematic review and meta-analysis
Source: Eur J Med Res. 2025 Dec 11;31:84. doi: 10.1186/s40001-025-03427-0 (PMC12801843; doi:10.1186/s40001-025-03427-0)
Supplement: Supplementary file 1 — Supplementary material 1: Table S2: Risk of Bias Assessment. Table S3: Risk of Bias Assessment. Table S4: Risk of Bias Assessment. [file 40001_2025_3427_MOESM1_ESM.docx]

| **Database** |  | **Results** |
| --- | --- | --- |
| PubMed  Embase  Cochrane Library  Clinicaltrial.gov | ((((((((((((4,4'-Diaminophenyl Sulfone) OR (4,4' Diaminophenyl Sulfone)) OR (Sulfone, 4,4'-Diaminophenyl)) OR (DADPS)) OR (Diaminodiphenylsulfone)) OR (Diaphenylsulfone)) OR (Sulfonyldianiline)) OR (Disulone)) OR (Avlosulfone)) OR (Sulfona)) OR (Dapson-Fatol)) OR (Dapsoderm-X)) AND (((((((((((((((((((((((((((((((Autoimmune Thrombocytopenia) OR (Autoimmune Thrombocytopenias)) OR (Thrombocytopenia, Autoimmune)) OR (Thrombocytopenias, Autoimmune)) OR (Immune Thrombocytopenic Purpura)) OR (Immune Thrombocytopenic Purpuras)) OR (Purpura, Immune Thrombocytopenic)) OR (Purpuras, Immune Thrombocytopenic)) OR (Thrombocytopenic Purpura, Immune)) OR (Thrombocytopenic Purpuras, Immune)) OR (Thrombocytopenic Purpura, Autoimmune)) OR (Immune Thrombocytopenia)) OR (Immune Thrombocytopenias)) OR (Thrombocytopenia, Immune)) OR (Thrombocytopenias, Immune)) OR (Werlhof Disease)) OR (Disease, Werlhof)) OR (Werlhof's Disease)) OR (Disease, Werlhof's)) OR (Werlhofs Disease)) OR (Autoimmune Thrombocytopenic Purpura)) OR (Autoimmune Thrombocytopenic Purpuras)) OR (Purpura, Autoimmune Thrombocytopenic)) OR (Purpuras, Autoimmune Thrombocytopenic)) OR (Idiopathic Thrombocytopenic Purpura)) OR (Idiopathic Thrombocytopenic Purpuras)) OR (Purpura, Idiopathic Thrombocytopenic)) OR (Purpuras, Idiopathic Thrombocytopenic)) OR (Thrombocytopenic Purpura, Idiopathic)) OR (Thrombocytopenic Purpuras, Idiopathic)) OR (Purpura, Thrombocytopenic, Autoimmune)) result  ((((((((((((4,4'-Diaminophenyl Sulfone) OR (4,4' Diaminophenyl Sulfone)) OR (Sulfone, 4,4'-Diaminophenyl)) OR (DADPS)) OR (Diaminodiphenylsulfone)) OR (Diaphenylsulfone)) OR (Sulfonyldianiline)) OR (Disulone)) OR (Avlosulfone)) OR (Sulfona)) OR (Dapson-Fatol)) OR (Dapsoderm-X)) AND (((((((((((((((((((((((((((((((Autoimmune Thrombocytopenia) OR (Autoimmune Thrombocytopenias)) OR (Thrombocytopenia, Autoimmune)) OR (Thrombocytopenias, Autoimmune)) OR (Immune Thrombocytopenic Purpura)) OR (Immune Thrombocytopenic Purpuras)) OR (Purpura, Immune Thrombocytopenic)) OR (Purpuras, Immune Thrombocytopenic)) OR (Thrombocytopenic Purpura, Immune)) OR (Thrombocytopenic Purpuras, Immune)) OR (Thrombocytopenic Purpura, Autoimmune)) OR (Immune Thrombocytopenia)) OR (Immune Thrombocytopenias)) OR (Thrombocytopenia, Immune)) OR (Thrombocytopenias, Immune)) OR (Werlhof Disease)) OR (Disease, Werlhof)) OR (Werlhof's Disease)) OR (Disease, Werlhof's)) OR (Werlhofs Disease)) OR (Autoimmune Thrombocytopenic Purpura)) OR (Autoimmune Thrombocytopenic Purpuras)) OR (Purpura, Autoimmune Thrombocytopenic)) OR (Purpuras, Autoimmune Thrombocytopenic)) OR (Idiopathic Thrombocytopenic Purpura)) OR (Idiopathic Thrombocytopenic Purpuras)) OR (Purpura, Idiopathic Thrombocytopenic)) OR (Purpuras, Idiopathic Thrombocytopenic)) OR (Thrombocytopenic Purpura, Idiopathic)) OR (Thrombocytopenic Purpuras, Idiopathic)) OR (Purpura, Thrombocytopenic, Autoimmune)) result  ("4,4'-Diaminophenyl Sulfone" OR "4,4' Diaminophenyl Sulfone" OR "Sulfone, 4,4'-Diaminophenyl" OR DADPS OR Diaminodiphenylsulfone OR Diaphenylsulfone OR Sulfonyldianiline OR Disulone OR Avlosulfone OR Sulfona OR "Dapson-Fatol" OR "Dapsoderm-X" OR Dapsone) in Title Abstract Keyword AND ("Autoimmune Thrombocytopenia" OR "Immune Thrombocytopenic Purpura" OR "Thrombocytopenic Purpura, Immune" OR "Werlhof Disease" OR "Autoimmune Thrombocytopenic Purpura" OR "Idiopathic Thrombocytopenic Purpura" OR "Immune Thrombocytopenia" OR "Thrombocytopenia, Immune" OR ITP) in Title Abstract Keyword - (Word variations have been searched)  ("Autoimmune Thrombocytopenia" OR "Immune Thrombocytopenic Purpura" OR "Thrombocytopenic Purpura, Immune" OR "Werlhof Disease" OR "Autoimmune Thrombocytopenic Purpura" OR "Idiopathic Thrombocytopenic Purpura" OR "Immune Thrombocytopenia" OR "Thrombocytopenia, Immune" OR ITP) \| ("4,4'-Diaminophenyl Sulfone" OR "4,4' Diaminophenyl Sulfone" OR "Sulfone, 4,4'-Diaminophenyl" OR DADPS OR Diaminodiphenylsulfone OR Diaphenylsulfone OR Sulfonyldianiline OR Disulone OR Avlosulfone OR Sulfona OR "Dapson-Fatol" OR "Dapsoderm-X" OR Dapsone) | 79  428  16  1 |

**Table S1: Search strategy**

| **Bias Domain** | **Risk Judgment** | **Justification** |
| --- | --- | --- |
| **1. Random sequence generation (selection bias)** | Low Risk | Randomized 1:1 allocation described; central allocation method likely used. |
| **2. Allocation concealment (selection bias)** | Low Risk | Not explicitly detailed, but study likely had proper allocation processes given IRB approval and multicenter RCT design. |
| **3. Blinding of participants and personnel (performance bias)** | High Risk | Open-label study; blinding not feasible due to visible dapsone side effects (e.g., stool discoloration, hemolysis). |
| **4. Blinding of outcome assessment (detection bias)** | Low Risk | Primary endpoint was objective (platelet count); independently assessed by a blinded expert. |
| **5. Incomplete outcome data (attrition bias)** | Low Risk | Intention-to-treat analysis used; dropouts were accounted for and included in outcome calculations. |
| **6. Selective reporting (reporting bias)** | Low Risk | All pre-specified endpoints (response rate, adverse events) reported; trial registration cited (NCT02627417, NCT02877706). |
| **7. Other sources of bias** | Unclear Risk | Early discontinuation (78.3% in dapsone group) may influence interpretation; potential influence from industry affiliations declared transparently. |

**Table S2: Risk of Bias Assessment (Larue et al.)**

| **Bias Domain** | **Risk Judgment** | **Justification** |
| --- | --- | --- |
| **1. Random sequence generation (selection bias)** | High Risk | No mention of randomization; study design appears observational or uncontrolled. |
| **2. Allocation concealment (selection bias)** | High Risk | No allocation process or control group included. |
| **3. Blinding of participants and personnel (performance bias)** | High Risk | No blinding reported; likely open-label. |
| **4. Blinding of outcome assessment (detection bias)** | Unclear Risk | Outcome assessors not mentioned; unclear if blinded to treatment. |
| **5. Incomplete outcome data (attrition bias)** | Low Risk | All patients accounted for; outcomes clearly reported. |
| **6. Selective reporting (reporting bias)** | Low Risk | Outcomes pre-specified in methods are fully reported in results. |
| **7. Other sources of bias** | High Risk | Small sample size, no comparator, retrospective design increase potential bias. |

**Table S3: Risk of Bias Assessment (TK Dutta 2001)**

|  | Selection | Comparability | Comparability | Outcome | Outcome | Outcome | Outcome |  |  | Study | Representativeness of the exposed cohort | Selection of the non-exposed cohort | Ascertainment of exposure |
| --- | --- | --- | --- | --- | --- | --- | --- | --- | --- | --- | --- | --- | --- |
| Ascertainment of exposure | Demonstration that outcome of interest was not present at the start of study | Controls for the most important risk factors | Controls for other risk factors | Controls for other risk factors | Assessment of outcome | Assessment of outcome | Was follow up long enough for outcomes to occur | Adequacy of follow up of cohorts | Total quality score | Esteve C, Samson M | 1 | 0 | 1 |
| 1 | 1 | 0 | 0 | 0 | 1 | 1 | 1 | 1 | 6 | Colella MP | 1 | 0 | 1 |
| 1 | 1 | 1 | 0 | 0 | 1 | 1 | 1 | 1 | 7 | None | None | None | None |

**Table S4: Risk of Bias Assessment (New Castle Ottawa Scale)**

| **Study** | **Year** | **Study Design** | **Total patients** | **Any adverse events (n, %)** | **Specific adverse events (counts / notes)** | **Discontinued due to AEs (n, %)** |
| --- | --- | --- | --- | --- | --- | --- |
| Larue et al. | 2024 | Randomized controlled trial (dapsone + prednisone arm) | 46 | 36 discontinued; multiple SAEs reported (n=3) | Hemolytic anemia: 21 events (45.6% of events); 4 required treatment cessation (8.7%); Methemoglobinemia: 10 events (21.7%); 5 required cessation (10.9%); Drug-related skin reactions: 8 events (17.4%); 3 required cessation (6.5%); Other SAEs: 10 events (21.7%)/6 required cessation; 1 death (ICH). | 19 patients |
| Colella et al. | 2021 | Single-center retrospective cohort | 122 | Symptomatic adverse events in 16% (n reported in text) | Symptomatic hemolytic anemia: 5; Symptomatic methemoglobinemia: 5; GI symptoms: 4; Skin rash: 2; Pruritus: 1; Headache: 1; Hepatic toxicity: 1. Methemoglobin elevations in 45/54 tested (83%) (mostly mild). | Treatment interrupted due to side effects in 11% (n=13) |
| Estève et al. | 2017 | Retrospective monocentric study | 42 | Side effects in 31% (n=13) | Skin rash: 5 (including 2 sulfone syndrome/DRESS); Methemoglobinemia: 4; Neuropathy: 3; Dyspnea: 1; Fatigue: 1; Diarrhoea: 1. Side effects resolved in all but one (neuropathy). | Withdrawn due to side effects in 9 patients (22%) |
| Dutta 2001. | 2001 | A Prospective phase 1 & 2 | 8 | 1 | N/R | N/R |

**Table S5: Adverse events per study**
